# A Surgical Technique to Preserve the Subvalvular Apparatus in Patients Undergoing Mitral Valve Replacement for Severe Ischemic Regurgitation

**DOI:** 10.21470/1678-9741-2021-0320

**Published:** 2022

**Authors:** Jacob Zeitani, Ermal Likaj, Saimir Kuci, Antonio Pellegrino

**Affiliations:** 1 Department of Neuroscience and Rehabilitation, University of Ferrara, Ferrara, Italy.; 2 Department of Cardiac Surgery, University Hospital Center “Mother Teresa”, Tirana, Albania.; 3 Department of Cardiac Surgery, University of Rome Tor Vergata, Rome, Italy.

**Keywords:** Bioprosthesis, Emergencies, Heart Ventricles, Mitral Valve Insufficiency, Myocardial Revascularization

## Abstract

Severe functional mitral valve regurgitation should be treated in patients undergoing myocardial revascularization. When replacement is considered the best therapeutic option, preservation of the mitral subvalvular apparatus is crucial, especially in the emergency setting, because of its primary role in preserving geometry and function of left and right ventricles. Here we present a simple and quick technique, where subvalvular apparatus is preserved in toto in patients undergoing mitral valve replacement with a bioprosthesis.

**Table:** 

Abbreviations, Acronyms & Symbols			
AF	= Atrial fibrillation	ICU	= Intensive care unit
AL	= Anterior leaflet	LVEF	= Left ventricular ejection fraction
CABG	= Coronary artery bypass grafting	LVOTO	= Left ventricular outflow tract obstruction
COPD	= Chronic obstructive pulmonary disease	MI	= Myocardial infarction
CPB	= Cardiopulmonary bypass	MR	= Mitral regurgitation
CRF	= Chronic renal failure	MVR	= Mitral valve replacement
EDV	= End-diastolic volume	NYHA	= New York Heart Association
ESV	= End-systolic volume	PMR	= Papillary muscle rupture
FMR	= Functional mitral regurgitation	TEE	= Transesophageal echocardiogram
FU	= Follow-up		

## INTRODUCTION

The superiority of mitral valve repair over mitral valve replacement (MVR) when valvular incompetence is caused by degenerative disease is well established, and it is considered the procedure of choice. In contrast, in the functional mitral regurgitation (FMR), it is still under debate whether repair is better than replacement, especially when looking at long-term results. Indeed, approximately 50% of the patients who underwent mitral valve repair for FMR experienced moderate to severe grade of valve incompetence in a long-term follow-up^[1]^. Acute and severe valve regurgitation, although rare, can be the consequence of post infarction papillary muscle rupture (PMR), resulting in pulmonary edema, compromised hemodynamic status, and acute heart failure. In these cases, current clinical guidelines recommend urgent surgical treatment, because mortality rates are very high with conservative medical treatment only (50% and 80% at 24 hours and at one week, respectively)^[2]^.

Also, in this clinical setting, there is no consensus regarding the surgical approach. Several authors have proposed different mitral valve repair techniques reporting good intraoperative results and emphasizing the superiority of repair over replacement. To achieve such results, surgeons must be skilled with mitral valve repair and when considering the urgent-emergency setting, however, they may not feel comfortable with the long and elaborate repair procedure itself. Also, in the acute forms, often the size of the left atrium is normal, allowing limited view and surgical field, making repair of the subvalvular apparatus in appropriate manner more complex. Once replacement is considered, the subvalvular apparatus should be preserved, preferably of both leaflets. In a systematic review, Athanasiou et. al.^[3]^ could demonstrate better early and mid-term results when the mitral valve apparatus had been preserved. Ozdemir et al.^[4]^ evaluated the clinical outcomes in patients who underwent MVR and found that preservation of both leaflets appears to guarantee better results when compared to preservation of the posterior leaflet only. However, in spite of the reported advantages of preservation of the subvalvular apparatus of both leaflets, anterior leaflet (AL) resection is often performed in order to avoid systolic anterior motion and left ventricular outflow tract obstruction (LVOTO)^[5,6]^. To avoid such complication while preserving the AL, different surgical techniques have been proposed, some of which being complex and time-consuming. Furthermore, other complications related to such procedures, including PMR and embolization, have also been reported^[6]^. Considering the emergency setting, surgical and aortic cross-clamping times should be limited, balancing risks with benefits to prevent low cardiac output at the end of the procedure^[7]^.

**Fig. 1:**
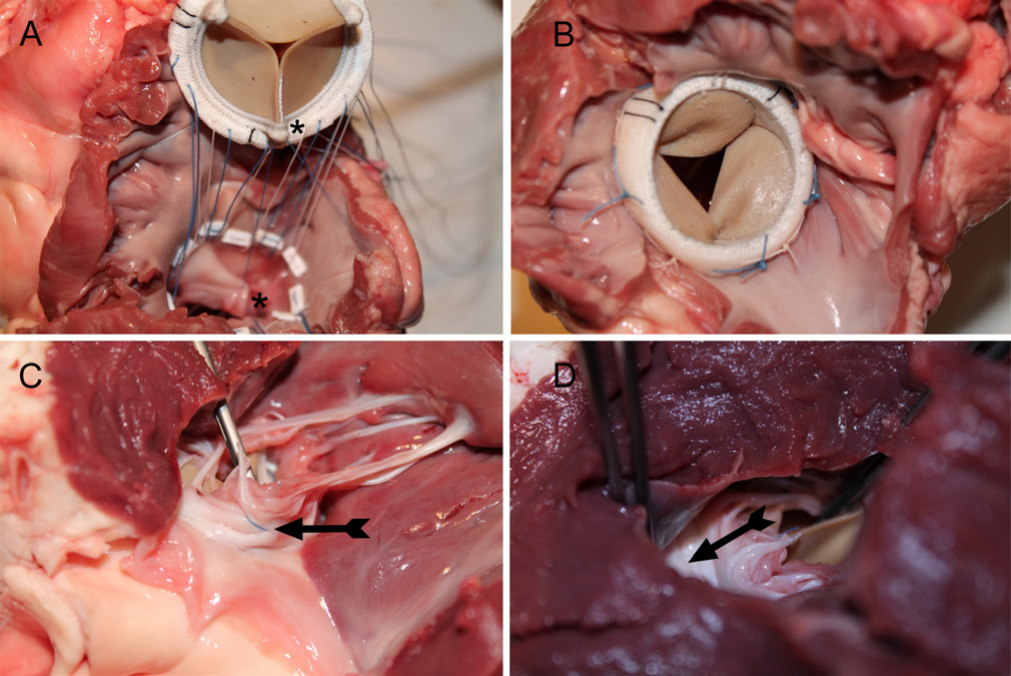
Images of a step-by-step anterior mitral leaflet anchoring using a fresh swine heart for illustration. A) Polyester sutures positioned in the mitral annulus and in the prosthesis cuff (those in correspondence to the anterior leaflet) and a 4-0 polypropylene suture passed in the anterior leaflet and in the polyester material of the bioprosthesis strut (*). B) Complete positioning of the prosthesis in the mitral orifice. C) Left ventricular view of the anchoring polypropylene stitch (arrow) and complete subvalvular apparatus. D) Free left ventricular outflow tract (arrow).

We describe a simple and quick surgical technique, in which the valve apparatus is preserved in toto, and the anterior mitral leaflet is anchored directly to the bioprosthesis strut, thus avoiding leaflet migration into the outflow tract. For better illustration of this technique, we performed this procedure on a fresh swine heart as shown in Figure 1.

## TECHNIQUE

All procedures were performed via median sternotomy, aortic and bicaval cannulation, and with antegrade warm-blood hyperkalemic cardioplegia for myocardial protection. Following revascularization of the inferior and lateral walls, when indicated, the mitral valve was exposed through a left atriotomy, performed parallel to the interatrial groove. For the MVR, the mitral orifice was measured, and reinforced 2-0 polyester sutures were placed in standard manner. Then, in order to anchor the AL to the bioprosthesis strut, a 4-0 polypropylene suture was used, first passed at the distal edge of the AL (A2 segment), then both needles were passed in parallel along the strut (a couple of stitch bites), and finally (passing from the ventricular aspect to the atrial one) in the prosthesis cuff (Figure 1A). At the end, the polyester sutures were passed in the prosthesis cuff, and once the prosthesis was tied to the annulus, the anchoring polypropylene suture was pulled to attach the edge of the leaflet to the strut and knotted (Figures 1B and C).

The procedure was performed successfully in five patients who underwent myocardial revascularization and MVR for severe FMR; one was an emergency case, a patient who experienced PMR following myocardial infarction, and the other four were consecutive elective cases. Patients’ characteristics are reported in Table 1, and intra and postoperative data in Table 2. The leaflet anchoring procedure required a few minutes in all cases (2-3 min). At the end of the procedure, intraoperative transesophageal echocardiogram (TEE) revealed normal prosthesis function and no LVOTO. Follow-up echocardiography up to 30 (mean 17±9) months was performed in all patients, confirming normal prosthesis function and no AL migration. Postoperative and at follow-up echocardiography control showed improvement in left ventricular ejection fraction (LVEF) and diameters (Table 3).

**Table 1:** Patients’ characteristics.

Variables	
Age (years)	71±4.3
Male	4
Preoperative NYHA dyspnea class	
III	1
IV	4
Angina class	
3	3
4	0
**MI**	**5**
**LVEF (%)**	**43±15**
**MR**	
**3+**	**2**
**4+**	**3**
Diabetes	3
COPD	5
CRF	2
Hypertension	4

COPD=chronic obstructive pulmonary disease; CRF=chronic renal failure; LVEF=left ventricular ejection fraction; MI=myocardial infarction; MR=mitral regurgitation; NYHA=New York Heart Association

**Table 2:** Intraoperative and postoperative data.

Variables	No.
CABG (patients)	5
Mean grafts/patient	2.6±0.8
Prosthesis size	
27 mm	1
29 mm	3
31 mm	1
CPB time (min)	94±48
Aortic cross-clamping time (min)	72±26
Intubation time (hours)	7±4
ICU stay (hours)	36±20
Postoperative AF	2
Postoperative in-hospital stay (days)	7.5±3

AF=atrial fibrillation; CABG=coronary artery bypass grafting; CPB=cardiopulmonary bypass; ICU=intensive care unit

**Table 3:** Echocardiographic data.

Variables	Preoperative	Postoperative	FU
LVEF (%)	43±15	47±9	50±4
MR		_	_
3+	2	_	_
4+	3	_	_
EDV (ml)	153±48	149±56	131±61
ESV (ml)	120±41	98±55	95±62

EDV=end-diastolic volume; ESV=end-systolic volume; FU=follow-up; LVEF=left ventricular ejection fraction; MR=mitral regurgitation

## DISCUSSION

Mitral valve repair is believed to be the procedure of choice to treat degenerative mitral valve regurgitation^[8]^. However, which is better, either valve repair or replacement is still under debate in the FMR disease and, in particular, when PMR has occurred after myocardial infarction. Current guidelines do not indicate which of the procedures is superior. Recent randomized trials suggest that MVR may be superior to mitral valve repair for the treatment of ischemic mitral regurgitation when recurrence of regurgitation is considered^[1]^.

Preservation of the AL and its subvalvular apparatus during MVR is believed to preserve left ventricular function by maintaining an annular-papillary connection and avoiding left ventricular deformation. It also reduces left ventricular chamber size and systolic afterload as compared to the effects of the conventional procedure of excision of the entire subvalvular apparatus^[3]^. In a prospective study, Yun et al.^[9]^ could demonstrate that anterior chordal transection resulted in a significant impairment of global and regional LVEF and, more importantly, in deleterious effects in terms of a significant decrease in right ventricular function. However, despite the striking evidence advocating preservation of the subvalvular apparatus, these conservative procedures are not performed routinely. Surgeons are reluctant to use preservation techniques as it is argued that the preserved tissue reduces the mitral orifice area, thus a smaller prosthesis must be used, and LVOTO might occur especially in patients with septal hypertrophy^[5]^. Furthermore, some of the reported AL preservation techniques are complex, time-consuming, and may cause alterations of the left ventricular geometry, PMR, and systemic embolization^[3]^. Total extracorporeal circulation time and, more importantly, aortic cross-clamping time should be taken into consideration when combined procedures are performed, especially when patients with poor left ventricular function are treated and/or when surgery is performed in an emergency setting^[7]^.

Miki et al.^[10]^ proposed partial detachment and division of the AL in anterior e posterior segments and reattachment of the chordae at the level of the corresponding commissures. However, the authors emphasize the need to inspect leaflets and subvalvular apparatus and, in particular, the risk of interference with the normal leaflet motion of a mechanical prosthesis, pointing out the importance of choosing a low-profile prosthesis to reduce such risk. Buffolo et al.^[11]^ described an innovative surgical technique for preservation of valve leaflets and subvalvular apparatus. In particular, the proposed technique includes AL detachment from the annulus, leaflet splitting (A2), and reattachment of the obtained two segments (A1 and A3) separately at the level of the corresponding anterior and posterior commissures. In this way, the papillary muscles are suspended with the aim of avoiding negative left ventricular remodeling. The study results suggest that patients in refractory heart failure with cardiomyopathy and ischemic mitral regurgitation might benefit from this technique.

Indeed, in consideration of the high incidence of recurrence of regurgitation following mitral valve repair in the FMR, especially in an emergency, MVR could be a better option; in this case, a bioprosthesis is more frequently indicated due to ageing of the population or short life expectancy of the patients. Moreover, only with implantation of a bioprosthesis, the subvalvular apparatus in toto can be preserved in a simple way as herein proposed.

**Fig. 2:**
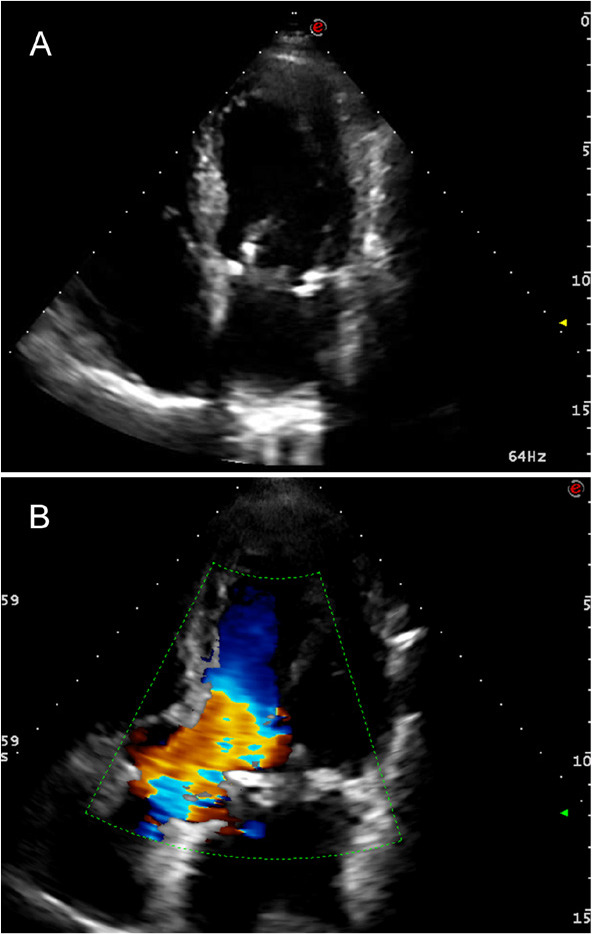
Postoperative transthoracic echocardiographic images demonstrating limited anterior leaflet motion (A), and echo doppler showing no subaortic valve gradient (B).

In fact, as far as our technique is concerned, the time required to immobilize AL was very short, and the procedure resulted to be very simple. When the mid portion of the anterior mitral valve leaflet is anchored to the prosthesis strut, migration of the leaflet, during the cardiac cycle, is avoided (Figures 1D and 2). Based on the leaflet morphology and size, the stitch can be tailored and applied to any level of the leaflet in order to achieve fixation without causing excessive tension. Indeed, AL relocation might lead to unbalanced stress on the chordae and the papillary muscle, with the risk of a new rupture, especially when surgery is performed shortly after myocardial infarction and left ventricular remodeling is still evolving. De Canniere et al.^[5]^ described their technique in leaflet preservation, reporting delayed new PMR, apparently due to excessive stress of the plicated mitral leaflets.

However, to reduce the risk of tear of the attached leaflet, consistence of the valve tissue should also be taken into consideration especially if high traction is desired. In any case, large suture bites should be performed to guarantee optimal fixation.

## CONCLUSION

Obviously, the surgical technique that we propose has been employed in a number of cases that is too limited to allow a proper evaluation of its validity; the benefits of this approach should be further evaluated with a prospective randomized study. However, the simplicity and the short procedural time required to anchor the leaflet as well as the mid-term follow-up are encouraging.
